# Emerging Role of Circular RNAs in Hepatocellular Carcinoma Immunotherapy

**DOI:** 10.3390/ijms242216484

**Published:** 2023-11-18

**Authors:** Tasneem Abaza, Mostafa K. Abd El-Aziz, Kerolos Ashraf Daniel, Paraskevi Karousi, Maria Papatsirou, Sherif Ashraf Fahmy, Nadia M. Hamdy, Christos K. Kontos, Rana A. Youness

**Affiliations:** 1 Biology and Biochemistry Department, Molecular Genetics Research Team (MGRT), Faculty of Biotechnology, German International University (GIU), Cairo 11835, Egypt; tasneem.abaza11@gmail.com (T.A.); mostafakhaled8899@gmail.com (M.K.A.E.-A.); kerolos.daniel@gaf.ac (K.A.D.); 2 Biotechnology and Biomolecular Chemistry Program, Faculty of Science, Cairo University, Giza 12613, Egypt; 3 Biochemistry Department, Faculty of Pharmacy, Al-Azhar University, Assiut Branch, Assiut 71631, Egypt; 4 Biology and Biochemistry Department, Molecular Genetics Research Team (MGRT), School of Life and Medical Sciences, University of Hertfordshire Hosted by Global Academic Foundation, Cairo 11835, Egypt; 5 Department of Biochemistry and Molecular Biology, Faculty of Biology, National and Kapodistrian University of Athens, 15701 Athens, Greece; pkarousi@biol.uoa.gr (P.K.); papatsir@biol.uoa.gr (M.P.); 6 Department of Chemistry, School of Life and Medical Sciences, University of Hertfordshire Hosted by Global Academic Foundation, R5 New Garden City, New Capital, Cairo 11835, Egypt; sheriffahmy@aucegypt.edu; 7 Biochemistry Department, Faculty of Pharmacy, Ain Shams University, Cairo 11566, Egypt; nadia_hamdy@pharma.asu.edu.eg

**Keywords:** circular RNAs (circRNAs), hepatocellular carcinoma (HCC), immunotherapy, cytotoxic T lymphocytes, natural killer cells, tumor microenvironment (TME)

## Abstract

Hepatocellular carcinoma (HCC) is a highly fatal malignancy with limited therapeutic options and high recurrence rates. Recently, immunotherapeutic agents such as immune checkpoint inhibitors (ICIs) have emerged as a new paradigm shift in oncology. ICIs, such as programmed cell death protein 1 (PD-1) inhibitors, have provided a new source of hope for patients with advanced HCC. Yet, the eligibility criteria of HCC patients for ICIs are still a missing piece in the puzzle. Circular RNAs (circRNAs) have recently emerged as a new class of non-coding RNAs that play a fundamental role in cancer pathogenesis. Structurally, circRNAs are resistant to exonucleolytic degradation and have a longer half-life than their linear counterparts. Functionally, circRNAs possess the capability to influence various facets of the tumor microenvironment, especially at the HCC tumor–immune synapse. Notably, circRNAs have been observed to control the expression of immune checkpoint molecules within tumor cells, potentially impeding the therapeutic effectiveness of ICIs. Therefore, this renders them potential cancer-immune biomarkers for diagnosis, prognosis, and therapeutic regimen determinants. In this review, the authors shed light on the structure and functional roles of circRNAs and, most importantly, highlight the promising roles of circRNAs in HCC immunomodulation and their potential as promising biomarkers and immunotherapeutic regimen determinants.

## 1. Introduction

Liver cancer ranks sixth in primary neoplasm prevalence. In comparison to other malignancies, liver cancer has an overwhelming impact marked by a high fatality rate. Global statistics expect liver cancer incidence to exceed one million cases by 2025 [1]. Hepatocellular carcinoma (HCC) predominates, making up 80–90% of primary liver malignancies [2], with a bleak five-year survival rate of 18% [3].

HCC management is intricate due to its diverse presentation in an inflamed liver, often compelling multidisciplinary teams to cooperate [4]. Surgical options offer a cure for early HCC; however, <20% of the patients receive a timely diagnosis [5]. Advanced-stage survival is recorded to be <10% in five years [6]. Unfortunately, more than 70% of HCC patients experience recurrence post-curative therapy [7,8]. Chemotherapy remained the primary form of HCC treatment despite all its complications, including chemoresistance and detrimental overall effects on the patients [9,10]. Yet, recent advances in understanding the molecular drivers of HCC have led to a new era of molecular targeting agents such as tyrosine kinase inhibitors (TKIs) and mammalian target of rapamycin (MTOR) inhibitors [11,12]. Nonetheless, TKIs have limited survival gains and associated intolerance, prompting innovative strategies.

In the field of oncology, immune checkpoint inhibitors (ICIs) are considered to be a novel and innovative strategy [13,14,15,16,17]. Their implementation has saved a lot of late-stage patients, especially patients with advanced unresectable HCC tumors [18,19]. ICIs brought a fundamental change, capitalizing on HCC’s immune pathogenicity [20]. Recent progress in understanding tumor–immune dynamics emphasizes ICIs targeting cytotoxic T-lymphocyte-associated protein 4 (CTLA-4) and programmed cell death protein 1 (PDCD1, also known as PD-1), which enhance therapeutic outcomes across several malignancies [16,21].

In 2017, nivolumab, a PD-1 inhibitor, was the first Food and Drug Administration (FDA)-approved monoclonal antibody (mAb) for second-line targeted therapy in the treatment of HCC. A year later, pembrolizumab, another PD-1 inhibitor, was added to the list. In 2020, the FDA approved two important combinational mAbs as immunotherapeutic regimens for HCC patients, which were nivolumab/ipilimumab and atezolizumab/bevacizumab [22,23]. In October 2022, tremelimumab/durvalumab became the most recently FDA-approved combinational mAb immunotherapeutic regimen for adult patients with advanced unresectable HCC [24]. It is worth noting that, nowadays, internationally endorsed guidelines adopt atezolizumab/bevacizumab as the first-line therapy for advanced, treatment-naïve HCC patients [24,25]. Recent studies (IMbrave150 trial) underscore atezolizumab/bevacizumab superiority over sorafenib in overall survival, progression-free survival, and patient-reported outcomes [26]. Although the impact of ICIs on survival is significant, they have also been linked to autoimmune-like side effects due to their ability to stimulate the immune system. These adverse effects are often expressed in the form of neurological toxicities, hepatotoxicity, and cardiotoxicity [27,28,29]. Therefore, we need a better understanding of the molecular mechanisms underlying therapeutic response.

The regulation of ICI pathways mediated by non-coding RNAs (ncRNAs) is a promising field of research regarding the probability of ICI toxicity. A substantial portion of the human genome undergoes transcription, yielding a diverse array of ncRNAs [30,31,32]. Within this spectrum, this discourse focuses on three key types—long non-coding RNAs (lncRNAs) [33,34], microRNAs (miRNAs) [35,36], and circular RNAs (circRNAs) [37]—that play fundamental roles in cancer pathogenesis [38,39,40].

In this review, the authors will focus on the most recently identified class of ncRNAs, which is circRNAs. circRNAs are closed-loop RNA structures, formed via the back splicing of precursor mRNA (pre-mRNA) molecules [41]. They are widely expressed in mammalian cells and known for stability, evolutionary conservation, and cell/tissue specificity. CircRNAs have diverse biological roles, including miRNA regulation, gene transcription modulation, RNA-binding protein (RBP) interaction, and protein/peptide encoding [42]. These functions primarily operate at epigenetic, transcriptional, and post-transcriptional levels [43,44]. CircRNAs regulate gene expression via an extended array of molecular mechanisms, influencing tumorigenesis and neoplastic progression [41]. Dysregulated circRNAs play pivotal roles in diseases, particularly in tumor development, influencing cell proliferation, apoptosis, and metastasis [45,46,47]. Most importantly, circRNAs have emerged as potent modulators of the tumor microenvironment (TME) and have a prospective role in tuning immunotherapeutic regimens’ efficiency and outcomes [16,39,48,49,50].

Coherently, the convergence of preclinical investigations consistently underscores the potential of manipulating ncRNAs to significantly potentiate the efficacy of immunotherapeutic interventions in the context of HCC. This scholarly review summarizes recent advancements in the landscape of circRNAs, followed by an in-depth exploration of the prospect of employing immunomodulatory circRNAs as plausible therapeutic targets/agents in HCC, accompanied by a comprehensive analysis of the intricate mechanistic frameworks that underlie these interactions.

## 2. Circular RNAs (CircRNAs)

### 2.1. What Are CircRNAs?

CircRNAs are a recently discovered class of ncRNA molecules. They are formed during the process of RNA transcript maturation. Structurally, circRNAs are covalently closed by a connection between a downstream donor and upstream acceptor RNA splice sites linked by a phosphodiester bond. CircRNAs were previously regarded as splicing junk but are now recognized as functional RNA molecules [31]. They have expression patterns that are particular to different tissues and cell types, and they are produced from a wide variety of genes [51]. It is noteworthy that circRNAs are implicated in biological processes that contribute to the development and spread of cancer [52,53].

Additionally, due to their circular shape and resistance to exoribonuclease activity, circRNAs have longer half-lives than their parental linear counterparts, making it possible to detect them even when produced at low levels [41,54,55]. For instance, exonic circRNAs are thought to be extremely stable in cells, with most circRNAs showing half-lives of over 48 h, as opposed to an average mRNA half-life of 10 h [54,56]. These characteristics imply that circRNAs could serve as useful biomarkers for the diagnosis and prognosis of cancer patients, as previously described in [41,50]. Yet, the current review focuses on the potential roles of circRNAs in modulating the HCC immunological profile and, thus, tuning the immune-suppressive TME in such chemo- and immuno-resistant tumors.

### 2.2. Biogenesis of CircRNAs

Recent research has shown that “back-splicing”, a type of pre-mRNA splicing, is responsible for the production of circRNAs [57]. CircRNAs have a distinctive closed-loop structure, created by linking a downstream 5′ splice donor site and an upstream 3′ splice acceptor site, in contrast to conventional pre-mRNAs with 5′ caps and 3′ polyadenylated tails [58,59].

**Figure 1:**
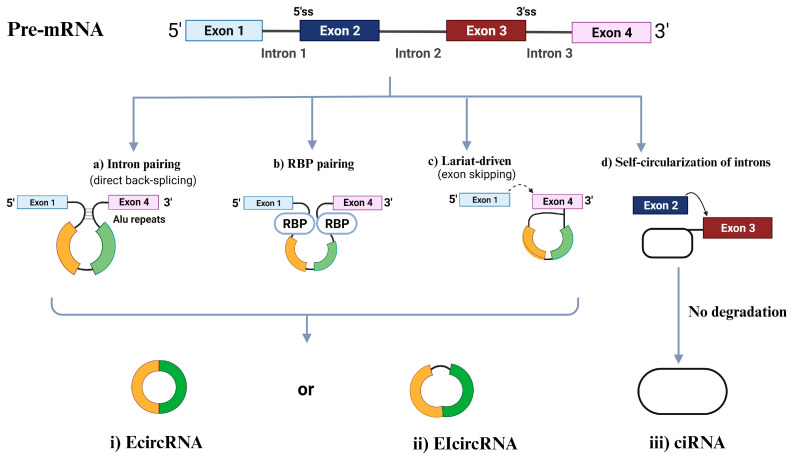
Circular RNA (circRNA) biogenesis. Schematic presentation of the different mechanisms of circRNAs biogenesis: intron painting (**a**), RBP pairing (**b**), Lariat driven (**c**), and the self-circularization of introns (**d**). ciRNA, intronic circRNA; EcircRNA, exonic circRNA; EIcircRNAs, exon-intron circRNA; RBP, RNA-binding protein; ss, splice site.

CircRNAs are primarily categorized into four types [60,61] based on the origin of their genomic regions: exonic circRNAs (EcircRNAs), retained-intron or exonic-intronic circRNAs (EIcircRNAs), intronic circRNAs (ciRNAs), and tRNA intronic circRNAs (tricRNAs) [62]. Over 80% of the circRNAs that have been discovered are EcircRNAs, and these circRNAs are mostly found in the cytoplasm [63,64]. As EcircRNAs sponge miRNAs and/or interact with RBPs, several studies have shown that EcircRNAs play significant roles in modulating the genetic expression of several coding transcripts [65,66]. EIciRNAs and ciRNAs, which compose a minor portion of circRNAs compared to EcircRNAs, are mostly found in the nucleus and, thus, can control the expression of their parental mRNAs, as shown in Figure 1 [57]. The following section will cover the four associated biogenesis mechanisms of circRNAs.

#### 2.2.1. Intron Pairing-Driven Circularization

The most frequent circularization process of EcircRNA and EIciRNA involves “direct back-splicing”, also known as intron-pairing-driven circularization, in which a particular pre-mRNA with ALU repeats is sheared to generate an EcircRNA or an EIciRNA following reverse-base complementary pairing [56].

#### 2.2.2. RBP-Induced Circularization

RBPs, which are thought to be trans-acting factors and include Quaking (QKI), Muscleblind (MBL), and Fused-in Sarcoma (FUS), may promote circularization by bridging similar intronic sequences [67]. The 3′ and 5′ termini of circularized exons can be brought into closer proximity through the dimerization of RBPs. This dimerization process also facilitates splicing by engaging with the sequences both upstream and downstream of the circularized exons [68].

#### 2.2.3. Lariat-Induced Circularization Driven by Spliceosomes

Lariat-driven circularization, also known as the exon-skipping mechanism, occurs as pre-mRNA partially folds during transcription. This folding brings the 5′ splice site (donor site) of the upstream intron close to the 3′ splice site (receptor site) of the downstream intron, forming a circRNA through back-splicing within the folded region. The remaining exons then combine to create a linear mRNA [56]. Moreover, back splicing can occur post-transcriptionally or co-transcriptionally, involving either a single exon or multiple exons with intervening introns [69].

#### 2.2.4. Self-Circularization of Introns

Intron self-circularization occurs when a pre-RNA contains 7 nucleotides (nt) of guanine (G) and uracil (U)-rich sequence close to one exon and an 11 nt cytosine (C)-rich sequence close to another exon. This allows the introns to avoid branching and degradation during splicing, resulting in a stable intronic lariat structure [57].

### 2.3. Functional Roles of CircRNAs

**Figure 2:**
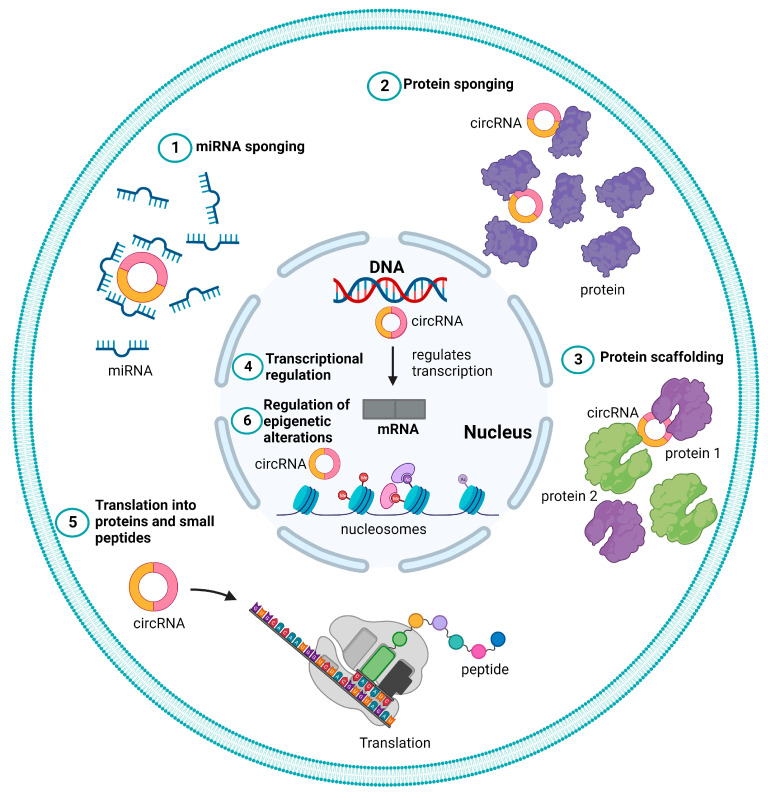
Different functional roles of circular RNAs (circRNAs). Schematic representation of different mechanisms of action of circRNAs represented as (1) microRNA (miRNA) sponge, (2) protein sponge or decoy (3) protein scaffolding, (4) transcriptional regulation, (5) translation to proteins, and peptide (6) regulation of epigenetic alterations.

CircRNAs typically function as regulatory ncRNA molecules, either directly by controlling gene transcription or indirectly by modifying other regulators, such as proteins and miRNAs. Further, the term “regulatory coding RNAs” refers to a subset of circRNAs that encode short functional peptides, as shown in Figure 2 and described below [53].

#### 2.3.1. miRNA Sponge

Some circRNAs may behave as miRNA sponges or sequesters because they include well-conserved canonical miRNA response elements (MREs) [70,71,72]. Some circRNAs that act as miRNA sponges can positively or adversely affect the expression of the corresponding targeted genes. Cerebellar degeneration-related protein 1 antisense (CDR1-AS or ciRS-7), a well-studied circRNA, has been linked to a variety of malignancies, including HCC and gastric cancer, as well as sponges miR-7 in embryonic zebrafish [73,74,75]. Indeed, a growing body of research has shown that the circRNA-miRNA-mRNA regulatory network may have significant effects on several diseases, including HCC [76,77].

For instance, circ-ZNF609 increases the expression of the myocyte-specific enhancer factor 2A (MEF2A), which improves vascular endothelial dysfunction by acting as an endogenous miR-615-5p sponge to decrease miR-615-5p activity [78]. Our previous work has also validated the tumor suppressor and immunomodulatory effects of miR-615-5p in HCC cell lines and primary natural killer (NK) cells isolated from HCC patients [79]. Thus, we highlighted that the potential activity of circ-ZNF609 in HCC patients deserves further investigation.

According to Zhong et al., circ-MYLK can ease the inhibition of its target vascular endothelial growth factor A (VEGFA), a crucial component of the VEGFA/VEGFR2/RAS/MAPK1 signaling pathway, in addition to being associated with the stage and grade of bladder carcinoma [80]. By sequestering miR-143 and increasing the production of its target *BCL2*, increased levels of circ-UBAP2 stimulate the proliferation of osteosarcoma cells while preventing apoptosis both in vitro and in vivo [81]. Similarly, circ-ABCB10 has been shown by Liang et al. to sponge miR-1271, promoting proliferation and inhibiting the apoptosis of breast cancer cells [82].

#### 2.3.2. Protein Sponge or Decoy

CircRNAs can also bind and sequester proteins using their protein-binding sites, functioning as an antagonist to impede their physiological function [83]. RBPs are one of the most common protein classes that can bind to circRNAs. For instance, circ-TNPO3 functions as a protein decoy for the insulin-like growth factor 2 mRNA binding protein 3 (IGF2BP3) to inhibit the capacity of gastric cancer cells to proliferate [84]. It is also worth noting that our previous work has highlighted the potent role of IGF2BPs in regulating HCC tumor activity and their potential regulation with miRNAs, including miR-1275 [85]. The expression of MYC proto-oncogene, as well as bHLH transcription factor (MYC) and its target, snail family transcriptional repressor 1 (SNAI1), is inhibited when circ-TNPO3 sequesters IGF2BP3, which reduces the ability of gastric cancer cells to proliferate and metastasize [84]. It was also reported that circ-SIRT1 binds to the eukaryotic translation initiation factor 4A3 (EIF4A3) in colorectal cancer cell lines, preventing its inhibitory impact on epithelial–mesenchymal transition and encouraging the proliferation and invasion of colorectal cancer cell lines [86].

CircRNAs can also decoy proteins by attaching themselves to cellular proteins and changing how they normally carry out their physiological functions [44,87]. Circ0000079 (ciR79) inhibits the induction of fragile X-related 1 (FXR1) protein and prevents its complexation with protein kinase C iota (PRKCI), thus preventing the FXR1/PRKCI-mediated phosphorylation of glycogen synthesis kinase 3β (GSK3B) and activator protein 1 (AP-1), suppressing SNAI1 protein levels and hindering non-small cell lung cancer growth [88].

#### 2.3.3. Protein Scaffolding

CircRNAs with enzyme and substrate binding sites are believed to serve as scaffolds that help two or more proteins to come into proximity and interact. CircFoxo3, which includes binding sites for MDM2 and p53, serves as an indicative case of this observation. In order to support the idea that circFoxo3 can serve as a protein scaffold, the mutation of these binding sites or circRNA silencing reduced the amount of p53 that an MDM2 antibody could pull down. Further research revealed that circFoxo3 promoted the ubiquitination of p53 by MDM2, which is then destroyed by the proteasome. Additionally, circACC1 forms a ternary complex with the regulatory β and γ subunits of AMP-activated protein kinase (AMPK), stabilizing and enhancing the enzymatic activity of the AMPK holoenzyme [89]. More circRNAs acting as scaffolds are expected to be identified in the future because of the longer half-lives of circRNAs [90].

#### 2.3.4. Transcriptional Regulation

CircSEP3 derived from *SEP3* exon 6 enhances the abundance of homologous exon 6-skipped variant by attaching to the host DNA locus and creating an RNA-DNA hybrid or R-loop, which causes transcription to pause and splicing factor recruitment [91]. Similarly, circSMARCA5 induces the expression of the shortened non-functional isoform by causing transcriptional termination of the SWI/SNF-related, matrix-associated, and actin-dependent regulator of chromatin, subfamily a, member 5 (*SMARCA5)* at exon 15 through R-loop formation [92]. EIciRNAs can interact with the U1 small nuclear ribonucleoprotein to increase the expression of parental genes through RNA-RNA interactions with snRNA molecules [93]. As lariats evade debranching, circRNAs can amass at their formation sites and enhance the activity of RNA polymerase II, resulting in the increased expression of the respective genes [57].

#### 2.3.5. Translation to Proteins and Peptides

The ability of circRNAs to undergo translation was originally discovered by Pamudurti et al. in 2017 [94]. According to bioinformatics studies, some circRNAs contain an open reading frame (ORF), which indicates that they can be translated. Ribosome profiling, which can sequence ribosome-covered RNAs to track translation in vivo, has shown convincing evidence that some circRNAs comprising internal ribosome entry sites (IRES) are translated based on an IRES-dependent mechanism [94], whereas other circRNAs are translated independently of IRES elements. The translation of circSHPRH into the SNF2 histone linker PHD RING helicase (SHPRH)-146aa protein was demonstrated to be IRES-dependent. It was discovered that SHPRH-146aa is a tumor suppressor protein that guards against the degradation of the SHPRH full-length protein. Therefore, incorrect circSHPRH translation affects tumor malignancy [95].

Other circRNAs have also been discovered to encode functional peptides and proteins that have tumor-promoting or -suppressing properties [95,96,97]. Finally, certain circRNAs can encode peptides without the need for IRES. Protein translation is made easier, for instance, by the m^6^A modification. The m^6^A reader protein YTH N6-methyladenosine RNA binding protein F3 (YTHDF3) interacts with translation initiation factors to start protein synthesis by binding to circRNAs that include m^6^A modification sites [98,99,100].

#### 2.3.6. Regulation of Epigenetic Alterations

Cancer commonly exhibits abnormal DNA methylation and histone alterations that are linked to the epigenetic regulation of gene expression [101,102]. It has been discovered that certain circRNAs control these epigenetic changes. According to Chen et al. [103], circFECR1 significantly reduced the amount of CpG DNA methylation in the promoter of Fli-1 proto-oncogene, ETS transcription factor (FLI1), which epigenetically activated FLI1. Through binding to the DNA methyltransferase 1 (DNMT1) promoter, circFECR1 has been shown to suppress the transcription of DNMT1, a crucial methyltransferase enzyme necessary for the upkeep of DNA methylation. Additionally, tet methylcytosine dioxygenase 1 (TET1) DNA demethylase might be attracted by circFECR1 to the FLI1 promoter and cause DNA demethylation. A component of polycomb-repressive complex 2 (PRC2), as an enhancer of zeste homolog 2 (EZH2), serves as an H3K27 methyltransferase and controls histone methylation [104,105]. Moreover, hsa-circ0020123 can upregulate EZH2 and zinc finger E-box binding homeobox 1 (ZEB1) using sponging miR-144, while circBCRC4 can enhance the expression of EZH2 by interacting with miR-101 [106,107].

### 2.4. Involvement of circRNAs in HCC Tumor Development and Progression

It has been reported that circRNAs have a fundamental role in the etiology of several human diseases, including several oncological conditions [108]. According to earlier investigations, circRNAs are thought to be important to the onset, development, and growth of HCC. For instance, circ0008450 induces HCC cellular proliferation, invasion, and migration and reduces apoptosis caused by sponging miR-548 [109]. Additionally, circRNA-104718 can similarly enhance HCC cellular proliferation, invasion, and migration and inhibit apoptosis by regulating the miRNA-218-5p/TXNDC5 axis [110]. The circular RNA hsa_circ_0078710 enhances cell proliferation by sequestering miR-31, resulting in the upregulation of histone deacetylase 2 (HDAC2) and cyclin-dependent kinase 2 (CDK2) expression [111]. Circ-ZEB1.33 facilitates the proliferation of HCC cells by modulating the miR-200a-3p/CDK6axis [112]. Hsa_circ_0016788 expedites HCC growth through the regulation of miR-481 and its downstream target cyclin-dependent kinase 4 (CDK4) [113]. Furthermore, hsa_circ_0091581 promotes the proliferation of HCC cells by elevating MYC levels, acting as a sponge for miR-526b [114]. Additionally, circBACH1 directly interacts with the RNA binding protein HuR, promoting the cytoplasmic accumulation of HuR, thus leading to decreased cyclin-dependent kinase inhibitor 1B (CDKN1B) expression [115], which influences cell cycle progression.

Pu et al. observed a significant increase in hsa_circ_0000092 expression in HCC tissues and cell lines. Depleting hsa_circ_0000092 inhibited HCC cell proliferation, migration, invasion, and angiogenesis in vitro and in vivo. This circRNA promotes HCC angiogenesis by acting as a miR-338-3p sponge, leading to increased expression of Jupiter microtubule-associated homolog 1 (JPT1), matrix metallopeptidase 9 (MMP9), and VEGFA [116]

Recent research highlights the pivotal roles of circRNAs in the regulation of apoptotic mechanisms within HCC. Specifically, these circRNAs target key components involved in both anti-apoptotic and pro-apoptotic signaling pathways. Notably, circ-BIRC6 exhibits significant overexpression in HCC tissue samples and correlates with the overall survival of HCC patients. Silencing circ-BIRC6 expression effectively enhances apoptosis in HCC cells by modulating BCL2 apoptosis regulator (BCL2) levels through the sequestration of miR-3918 [117]. Moreover, circ-0051443 displays reduced expression in HCC tissues and plasma. Exosomal circ-0051443 exerts a suppressive influence on the biological behaviors of HCC cells, primarily by promoting apoptosis through the interaction with miR-331-3p and the regulation of BCL2 antagonist/killer 1 (BAK1) [118]. On the other hand, certain circRNAs have inhibitory influences on the development of HCC. For instance, circADAMTS14 regulates miR-572/RCAN1, leading to the abrogation of HCC cellular hallmarks and inducing HCC cellular apoptosis machinery [119]; circRNA-5692 has a similar inhibitory impact on HCC progression by controlling the miR-328-5p/DAB2IP axis [120]. Table 1 represents a comprehensive list of all characterized oncogenic and tumor suppressor circRNAs in HCC.

## 3. Immunotherapy

Cancer immunotherapeutic modalities include several strategies, such as chimeric antigen receptor (CAR) T-cells, tumor vaccines, oncolytic viruses, and ICIs [174]. CAR T-cell therapy is a type of treatment through which a patient’s T cells are modified in a laboratory to attack cancer cells. This is performed by adding a gene for a particular receptor called a CAR to the T cells [49]. The modified CAR T cells are then grown in large numbers and infused back into the patient to kill tumor cells [175].

Cancer vaccine activates the body’s anti-tumor defenses by introducing tumor antigens. These antigens can be administered in various forms, such as whole cells, peptides, or nucleic acids. An ideal cancer vaccine aims to counteract the immune suppression present in tumors and stimulate both humoral and cellular immunity [176].

Oncolytic viruses are used as therapeutic agents to stimulate the selective destruction of tumor cells, allowing the targeted eradication of tumor cells while leaving normal tissues unaffected, and triggering anti-tumor immunity [177].

ICIs such as anti-CTLA4 and anti-PD-1 antibodies target immune checkpoint molecules present on immune cells to inhibit their activities, as previously described, thereby alleviating immunosuppression and prompting CD8+ T cells to eliminate cancerous cells in the body [178].

While the clinical outcomes of ICI appear to be promising, the overall level of response remains inadequate, as only 20% of individuals with solid tumors experience complete remission (CR) following treatment [179]. Such recent advancements in cancer immunotherapy and its combination regimens have greatly affected the HCC treatment outcomes, and clinical studies are continuing to pave the way for leveraging the additional benefits for HCC patients.

### 3.1. HCC Immunotherapy

HCC patients who solely depend on surgery, chemotherapy, or radiotherapy not only have low chances of survival but also do not experience significant improvements in their quality of life [12,18,85,180]. However, the introduction and rapid advancements in cancer immunotherapy, as described earlier, have provided increasingly promising results [79,181]. The presence of immune cells within the TME is crucial to fighting against tumors. However, cancerous cells can avoid the immune system and establish a complex equilibrium wherein diverse types of immune cells may contribute to the advancement of the tumor, spread to other parts of the body, and show resistance to treatment. Novel immunotherapy strategies are focused on reinstating the original equilibrium and enhancing the immune response against cancer through various means [182].

Immunotherapeutic regimens have successfully increased the survival rates, minimizing the side effects and providing long-term cancer control in advanced unresectable HCC patients [183]. Yet, a reliable marker for selecting the patients who would benefit the most from HCC immunotherapeutic regimens and those who would exhibit severe side effects is still missing [31,47].

Cell-free or circulating nucleic acids (CNAs) such as circRNAs in the blood have recently been identified as a new class of promising cancer–immune diagnostic/prognostic biomarkers to achieve the best outcome in HCC patients [31,184]. Prostate cancer-associated 3 (PCA3) has, in particular, been approved by the FDA and is currently being sold as Progensa by Hologic Gen-Probe (Marlborough, MA, USA) for prostate cancer diagnosis [185]. Circulating ncRNAs such as PCA3 are more reliable than other CNAs due to their high stability in the bloodstream and resistance to nuclease-mediated fragmentation, as extensively studied and reviewed by our research group in [186,187,188,189,190,191]. Plasma lncRNAs, in particular, were reported to be less sensitive to degradation induced via repetitive freeze–thaw cycles, as well as prolonged exposure to 45 °C and room temperature [192]. In this section of the review, the authors will shed light on the roles of circRNAs in HCC immunotherapy.

### 3.2. CircRNAs in HCC Immunotherapy

Since circRNAs have the potential to regulate many aspects of tumor immunity, they play a significant role in tumor immunotherapy; circRNAs have been observed to regulate the expression of immune checkpoint molecules in tumor cells, thereby allowing circRNAs to potentially hamper the therapeutic efficacy of ICIs [193]. Blocking the PD-1/PD-L1 checkpoint is one of the immunotherapeutic tactics widely used for treating various tumor types, including HCC [194].

CircRNAs in HCC can trigger immune system suppression and result in resistance to anti-PD-1 therapy. Evidence suggests that certain circRNAs can induce immune suppression and resistance against anti-PD-1 therapies in HCC. An illustrative case is circMET, an oncogenic immunosuppressor circRNA that triggers immune suppression through the SNAI1/DPP4/CXCL10 axis. Notably, sitagliptin, a dipeptidyl peptidase 4 (DPP4) inhibitor, augments CD8+ T cell infiltration in HCC tissues in diabetic individuals, potentially enhancing PD-1 blockade-based immunotherapy in selected HCC patients [195]. Therefore, the use of a DPP4 inhibitor or circMET siRNAs may significantly improve the effectiveness of immunotherapy with PD-1 blockade for the studied group of HCC patients.

**Figure 3:**
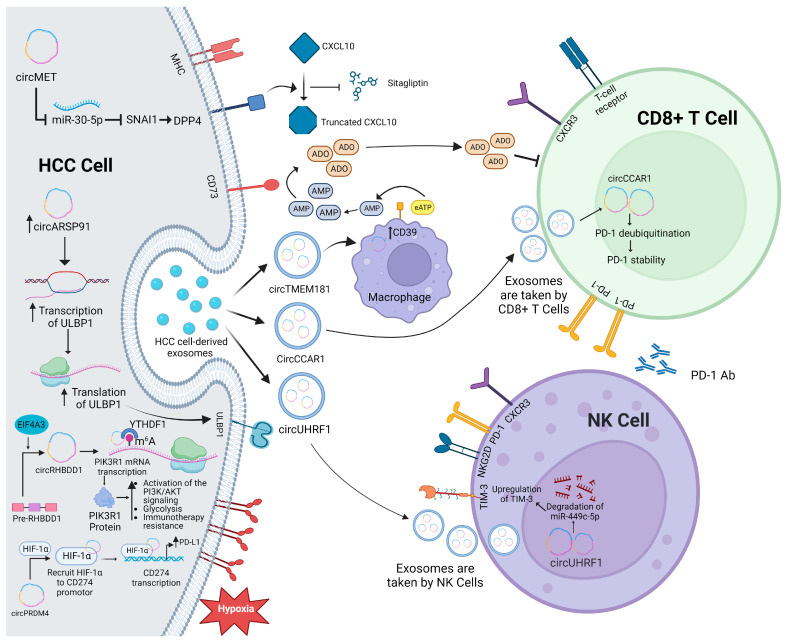
A snapshot of potential circRNAs as promising modulators of tumor microenvironment in HCC. Ab, antibody; ADO, adenosine; AKT, AKT serine/threonine kinase 1; AMP, adenosine monophosphate; CD39, ectonucleoside triphosphate diphosphohydrolase 1; CD73, 5′-nucleotidase ecto; CD8, cluster of differentiation 8; CXCL10, C-X-C motif chemokine ligand 10; CXCR3, C-X-C motif chemokine receptor 3; DPP4, dipeptidyl peptidase 4; eATP, extracellular adenosine triphosphate; EIF4A3, eukaryotic translation initiation factor 4A3; HCC, hepatocellular cancer; HIF-1a, hypoxia inducible factor 1 subunit alpha; M^6^A, N6-methyladenosine; MHC, major histocompatibility complex; NK, natural killer; NKG2D, killer cell lectin-like receptor K1; PD-1, programmed cell death protein 1; PD-L1, CD27/programmed cell death protein 1 ligand; PI3K, phosphoinositide 3-kinase; PIK3R1, phosphoinositide-3-kinase regulatory subunit 1; RHBDD1, rhomboid domain containing 1; Snail, snail family transcriptional repressor 1; TIM-3, hepatitis A virus cellular receptor 2; ULBP1, UL16 binding protein 1; YTHDF1, YTH N6-methyladenosine RNA binding protein F1.

CircPRDM4 is another immune-suppressor circRNA that works via a direct modulatory effect on the immune checkpoint, namely PD-L1 expression on HCC cells. CircPRDM4 induces the elevation of PD-L1 expression and facilitates the recruitment of HIF-1α onto the CD274 promoter under hypoxic conditions, resulting in CD8+ T cell-mediated immune evasion, as shown in Figure 3 [196]. This suggests that circPRDM4 is a promising onco-immune target in HCC. The presence of circRHBDD1 hinders the effectiveness of anti-PD-1 therapy in individuals with HCC. In HCC patients who respond to anti-PD-1 treatment, circRHBDD1 is found to be significantly elevated. However, when circRHBDD1 is targeted, the efficiency of anti-PD-1 therapy is enhanced in an immune-competent mouse model [197].

Additionally, it was reported that the overexpression of exosomal circTMEM181 secreted by tumor cells could impede the effectiveness of anti-PD-1 therapy in HCC and promote immunosuppression by upregulating the expression of CD39. Additionally, it hinders the ATP-adenosine pathway by targeting CD39 on macrophages. Conversely, rescuing the resistance to anti-PD-1 therapy in HCC can be achieved by targeting CD39 on macrophages [198]. Lastly, HCC-derived circCCAR1 was also reported to have an unfavorable effect on TME by inducing the permeant cellular dysfunction of CD8+ T-cells, and it is, thus, considered one of the most deleterious immune-evasion tactics orchestrated by HCC tumors (Figure 3). CircCCAR1 also plays a role in causing resistance to anti-PD-1 immunotherapy, suggesting a potential onco-immune target for HCC patients [199].

Regulatory T cells have the potential to disrupt the immune microenvironment of tumors and encourage immune evasion by suppressing the activation of effector T cells, such as CD4+ and CD8+ T cells [200,201]. Huang et al. conducted a study revealing that T cells can take up exosomes containing circGSE1. This uptake process plays a crucial role in enhancing the differentiation of CD4+ T cells into regulatory T cells by activating the miR-324-5p/TGFB1/SMAD3 pathway. The expansion of regulatory T cells, in turn, contributes to the increased proliferation, migration, and invasion of HCC. Consequently, exosomal circGSE1 holds promising potential as a target for immunotherapy [202].

NK cells play a crucial role in HCC TME, from the adaptive immune to the innate immune arm. Thus, the augmentation of NK cell infiltration and functionality increases patient survival across the spectrum of HCC patients [18]. This finding provides a novel vantage point for manipulating NK cell activity to augment the responsiveness of immunotherapeutic regimens among HCC patients [79,203]. Noteworthy investigations have unveiled instances of NK cell dysfunction within the HCC context, although the precise underpinnings of this phenomenon remain ambiguous. Zhang and collaborators have proffered insights into the mechanistic terrain, delineating how circUHRF1 orchestrates HCC progression and immune repression via an exosome-mediated and NK cell-dependent modality [127]. Mechanistically, circUHRF1 engenders NK cell dysfunction by sequestering miR-449c-5p, thus fostering hepatitis A virus cellular receptor 2 (HAVCR2, also known as TIM-3) expression. Significantly, circUHRF1 potentiates resistance to anti-PD-1 immunotherapy. Therefore, the strategic targeting of circUHRF1 emerges as a promising path for enhancing the therapeutic potency of anti-PD-1 immunomodulation in the HCC domain [127]. Moreover, Ma et al. utilized a particular plasmid to induce the overexpression of circARSP91 and then investigated how HCC cells would respond to NK cell cytotoxicity. They discovered that the UL16 binding protein 1 (ULBP1) showed a increased expression level, suggesting its potential influence on activating NK cells. Ultimately, their findings led them to conclude that circARSP91 can enhance the cytotoxicity of NK cells acting against tumors by upregulating ULBP1 (Figure 3) [204].

## 4. Conclusions and Future Perspectives

In conclusion, this review focuses on circRNAs as a novel investigational course of treatment in the field of tumor immunotherapy, highlighting the promising roles of circRNAs in the context of immunomodulation, which holds encouraging potential for improving the survival rates and outcomes of HCC patients. The authors shed light on the biogenesis of circRNAs, their functional modes, and their roles in HCC development and progression. However, special focus was given to summarizing the current literature discussing the roles of circRNAs as potential regulators of the immunogenic profile of HCC cells, cytotoxic T cells, and NK cells at the TME. Also, the review shed light on the promising roles of circRNAs as messengers between cancer and immune cells at the cancer–immune synapse at the TME. Yet, the authors also highlighted the gap in the literature concerning the mechanistic roles of circRNAs as possible modulators of other immune cells present at the TME, such as tumor-associated macrophages, tumor-associated fibroblasts, dendritic cells, and T regulatory cells. Nonetheless, the roles of circRNAs in regulating the immune-suppressive cytokine storm surrounding the tumor at the TME are under-investigated in HCC. This review paves the way for future possible usage of circRNAs to potentially offer a novel approach to enhance anti-tumor immune responses and overcome the challenges associated with current immunotherapeutic approaches available for HCC patients.

## Figures and Tables

**Table 1:** Oncogenic and tumor suppressor circular RNAs in hepatocellular carcinoma (HCC).

Circular RNA	Class	Molecular Targets	In Vitro/In Vivo/Ex Vivo Model	References
SCD-circRNA2	Oncogenic	MAPK1, RBM3	Huh7 HepG2 HCT-15 NCI-N87	[121]
circRHOT1	Oncogenic	NR2F6	HCC Tissues	[122]
circ-100338	Oncogenic	MMP2, MMP9	Hep3B HLE Huh7 BEL7402 SMCC7721 MHCC97L MHCC97H HCCLM3 HCCLM6	[123]
circ-0000092	Oncogenic	miR-338-3p	Hep3B LM3 MHCC97L SK-hep1 HepG2	[116]
circPRMT5	Oncogenic	miR-188-5p/HK2 axis	HCC tissues HCCLM3 SNU-387	[124]
circMAT2B	Oncogenic	PKM2	HepG2 Huh7 SMMC-772 MHCC-97L MHCC-97H	[125]
circASAP1	Oncogenic	MAPK1	MHCC97L MHCC97H HCCLM3	[126]
circβ-catenin	Oncogenic	β-catenin	Huh7	[97]
circUHRF1	Oncogenic	UHRF1	HepG2 HCCLM3 SMMC-7721 Huh 7 PLC/PRF/5 Hep3B	[127]
circ-CDYL	Oncogenic	PI3K-AKT-MTORC1/β-catenin and NOTCH2	HCCLM SMMC7721	[128,129]
circ-0046600	Oncogenic	HIF-1α	HepG2 SK-HEP-1	[130]
hsa_circ_0101432	Oncogenic	MAPK1	Huh-7 SK-HEP-1 HepG2 HLE	[131]
circMAN2B2	Oncogenic	MAPK1	HL-7702	[132]
circPTGR1	Oncogenic	MET	HepG2 97L LM3	[133]
circ-DB	Oncogenic	miR-34a, and USP7	HepG2 Hepa 1-6 3T3L1	[134]
circRNA Cdr1as	Oncogenic	AFP	SMMC-7721 Bel-7402 HepG2 Hep3B Huh-7 HB611	[135]
circRNA PVT1	Oncogenic	miR-203/HOXD3 pathway	SMMC-7721 Huh-7	[136]
circPVT1	Oncogenic	*TRIM23*/miR-377 axis	SNU-387 Huh-7	[137]
hsa_circ_0008450	Oncogenic	EZH2	SMMC7721 Sk-Hep-1 HepG2 Huh-7 HCCLM3	[138]
circ_0008450	Oncogenic	miR-548	HepG2 Huh-7, SMMC7721 Sk-Hep-1 HCCLM3	[109]
hsa_circRNA_103809	Oncogenic	miR-377-3p/FGFR1/MAPK1 axis	MHCC97L Huh7 SK-HEP-1 Hep3B HCCLM3	[139]
circRNA-104718	Oncogenic	miR-218-5p/TXNDC5	HCC nude mice model	[110]
circMYLK	Oncogenic	miR-362-3p/Rab23	Huh7 Hep3B	[140]
circ-ZNF652	Oncogenic	miR-29a-3p/GUCD1 Axis	SNU-387 Huh-7	[141]
circ_0000267	Oncogenic	miR-646	HepG2 Huh-7 SMMC7721 Sk-Hep-1 HCCLM3	[142]
circ-FOXP1	Oncogenic	miR-875-3p, miR-421, SOX9 factor	SNU-387 HepG2 Hep3B Huh7 SMMC-7721 HCCLM3	[143]
circRNA_104075	Oncogenic	YAP-dependent tumorigenesis through regulating HNF4a	Bel-7402 SMMC-7721 Huh7 HepG2 Hep1 Bel-7404 THLE-3 HL-7702	[144]
hsa_circ_101280	Oncogenic	miR-375/JAK2	HepG2 SNU-398	[145]
circRNA-101368	Oncogenic	HMGB1/RAGE	HCCLM3 HepG2	[146]
circ-ZEB1.33	Oncogenic	miR-200a-3p-CDK6	97H Huh7 HepG2 SNU423 SNU475 L02	[112]
circFBLIM1	Oncogenic	miR-346	HCC tissues HCC mouse model	[147]
hsa_circ_0103809	Oncogenic	miR-490-5p/SOX2 signaling pathway	MHCC97H HepG2 Huh7 SMMC7721 SK-Hep1	[148]
hsa_circ_0016788	Oncogenic	miR-486/CDK4	HepG2 Hep3B Huh7 HCCLM3 MHCC97L	[113]
hsa_circRBM23	Oncogenic	miR-138	HCC tissues HepG2 Huh7 Bel-7402	[149]
hsa_circ_0005075	Oncogenic	miR-431	SMMC-7721	[150]
circABCC2	Oncogenic	miR-665	HepG2 Bel-7402 MHCC97H	[151]
hsa_circ_100338	Oncogenic	MTOR signaling pathway	SMMC7721 Bel-7402 Hep3B	[152]
circ_0091581	Oncogenic	miR-591/FOSL2 axis	THLE-2	[153]
circPCNX	Oncogenic	miR-506	HL-7702 SMMC-7721 HuH-7 Hep3B HepG2	[154]
hsa_circ_0056836	Oncogenic	miR-766-3p/FOSL2 axis	Huh7 HepG2 SNU449 SK-HEP-1	[155]
circ- HOMER1	Oncogenic	miR-1322 on CXCL6	Sk-Hep-1 SMMC7721 HCCLM3 Huh-7 HepG2	[156]
circ_0091579	Oncogenic	miR-136-5p/TRIM27 miR-1270/YAP1 miR-1225/PLCB1	HCCLM3 MHCC97H Huh-7	[157,158,159]
circ_0001955	Oncogenic	miR-516a-5p miR-646 miR-145-5p/NRAS	Huh-7 HepG2 SMMC-7721 Bel-7402 Hep-3B	[160,161,162]
circTRIM33-12	Tumor suppressor	miR-191	HCC tissues MHCC97-L MHCC97-H LM3	[163]
circHIAT1	Tumor suppressor	PTEN	Hep3B SMMC-7721 HepG2 LM3	[164]
circLARP4	Tumor suppressor	miR-761/RUNX3/p53/CDKN1A pathway	Huh7 Hep3B SMMC7721 HepG2	[165]
circMTO1	Tumor suppressor	miR-9-5p/NOX4 axis	HepG2 Hep3B	[166]
circITCH	Tumor suppressor	miR-184	Huh7 HCCLM3 SMMC-7721 MHCC97H HepG2	[167]
circFBXW4	Tumor suppressor	miR-18b-3p/FBXW7 axis	LX-2	[168]
mmu_circ_34116	Tumor suppressor	miR-661/PTPN11	HepG2, SNU449	[169]
hsa_circ_0007874/cMTO1	Tumor suppressor	miR-338-5p	HCCLM3 MHCC97-L Hep3B SMMC-7721 Huh7 Bel-7402 MHCC97-H	[170]
circ608	Tumor suppressor	miR-222/PINK1	Primary hepatic stellate cells (PHSCs) from C57BL/6 mice	[171]
hsa_circ_0070963	Tumor suppressor	miR-223-3p LEMD3	LX2	[172]
hsa_circ_0004018	Tumor suppressor	miR-626/DKK3	Huh7 Bel7402 SNU182 Hep3B SNU449	[173]

## Data Availability

Not applicable.

## References

[1] Llovet J.M., Kelley R.K., Villanueva A., Singal A.G., Pikarsky E., Roayaie S., Lencioni R., Koike K., Zucman-Rossi J., Finn R.S. (2021). Hepatocellular carcinoma. Nat. Rev. Dis. Primers.

[2] Ringelhan M., Pfister D., O’Connor T., Pikarsky E., Heikenwalder M. (2018). The immunology of hepatocellular carcinoma. Nat. Immunol..

[3] Siegel R.L., Miller K.D., Fuchs H.E., Jemal A. (2022). Cancer statistics, 2022. CA Cancer J. Clin..

[4] Youssef S.S., Abbas E., Youness R.A., Elemeery M.N., Nasr A.S., Seif S. (2022). PNPLA3 and IL 28B signature for predicting susceptibility to chronic hepatitis C infection and fibrosis progression. Arch. Physiol. Biochem..

[5] Bruix J., Sherman M., American Association for the Study of Liver D. (2011). Management of hepatocellular carcinoma: An update. Hepatology.

[6] Llovet J.M., Bustamante J., Castells A., Vilana R., Ayuso Mdel C., Sala M., Bru C., Rodes J., Bruix J. (1999). Natural history of untreated nonsurgical hepatocellular carcinoma: Rationale for the design and evaluation of therapeutic trials. Hepatology.

[7] Kumada T., Nakano S., Takeda I., Sugiyama K., Osada T., Kiriyama S., Sone Y., Toyoda H., Shimada S., Takahashi M. (1997). Patterns of recurrence after initial treatment in patients with small hepatocellular carcinoma. Hepatology.

[8] Youssef S.S., Youness R.A., Abbas E.A.E., Osman N.M., ELFiky A., El-Kassas M. (2022). miR-516a-3P, a potential circulating biomarker in hepatocellular carcinoma, correlated with rs738409 polymorphism in PNPLA3. Per Med..

[9] Lohitesh K., Chowdhury R., Mukherjee S. (2018). Resistance a major hindrance to chemotherapy in hepatocellular carcinoma: An insight. Cancer Cell Int..

[10] Miyahara K., Nouso K., Yamamoto K. (2014). Chemotherapy for advanced hepatocellular carcinoma in the sorafenib age. World J. Gastroenterol..

[11] Ferrin G., Guerrero M., Amado V., Rodriguez-Peralvarez M., De la Mata M. (2020). Activation of mTOR Signaling Pathway in Hepatocellular Carcinoma. Int. J. Mol. Sci..

[12] Youness R.A., El-Tayebi H.M., Assal R.A., Hosny K., Esmat G., Abdelaziz A.I. (2016). MicroRNA-486-5p enhances hepatocellular carcinoma tumor suppression through repression of IGF-1R and its downstream mTOR, STAT3 and c-Myc. Oncol. Lett..

[13] Abdel-Latif M., Youness R.A. (2020). Why natural killer cells in triple negative breast cancer?. World J. Clin. Oncol..

[14] El Din G.S., Youness R.A., Assal R.A., Gad M.Z. (2020). miRNA-506-3p Directly Regulates rs10754339 (A/G) in the Immune Checkpoint Protein B7-H4 in Breast Cancer. Microrna.

[15] Ramzy A., ElSafy S., Elshoky H.A., Soliman A., Youness R., Mansour S., Sebak A. (2022). Drugless nanoparticles tune-up an array of intertwined pathways contributing to immune checkpoint signaling and metabolic reprogramming in triple-negative breast cancer. Biomed. Mater..

[16] Selem N.A., Nafae H., Manie T., Youness R.A., Gad M.Z. (2023). Let-7a/cMyc/CCAT1/miR-17-5p Circuit Re-sensitizes Atezolizumab Resistance in Triple Negative Breast Cancer through Modulating PD-L1. Pathol. Res. Pract..

[17] Mekky R.Y., Ragab M.F., Manie T., Attia A.A., Youness R.A. (2023). MALAT-1: Immunomodulatory lncRNA hampering the innate and the adaptive immune arms in triple negative breast cancer. Transl. Oncol..

[18] Youness R.A., Rahmoon M.A., Assal R.A., Gomaa A.I., Hamza M.T., Waked I., El Tayebi H.M., Abdelaziz A.I. (2016). Contradicting interplay between insulin-like growth factor-1 and miR-486-5p in primary NK cells and hepatoma cell lines with a contemporary inhibitory impact on HCC tumor progression. Growth Factors.

[19] Soliman A.H., Youness R.A., Sebak A.A., Handoussa H. (2023). Phytochemical-derived tumor-associated macrophage remodeling strategy using Phoenix dactylifera L. boosted photodynamic therapy in melanoma via H19/iNOS/PD-L1 axis. Photodiagnosis Photodyn. Ther..

[20] Pinato D.J., Guerra N., Fessas P., Murphy R., Mineo T., Mauri F.A., Mukherjee S.K., Thursz M., Wong C.N., Sharma R. (2020). Immune-based therapies for hepatocellular carcinoma. Oncogene.

[21] Zhu A.X., Kang Y.K., Yen C.J., Finn R.S., Galle P.R., Llovet J.M., Assenat E., Brandi G., Pracht M., Lim H.Y. (2019). Ramucirumab after sorafenib in patients with advanced hepatocellular carcinoma and increased alpha-fetoprotein concentrations (REACH-2): A randomised, double-blind, placebo-controlled, phase 3 trial. Lancet Oncol..

[22] Saung M.T., Pelosof L., Casak S., Donoghue M., Lemery S., Yuan M., Rodriguez L., Schotland P., Chuk M., Davis G. (2021). FDA Approval Summary: Nivolumab Plus Ipilimumab for the Treatment of Patients with Hepatocellular Carcinoma Previously Treated with Sorafenib. Oncologist.

[23] Casak S.J., Donoghue M., Fashoyin-Aje L., Jiang X., Rodriguez L., Shen Y.L., Xu Y., Jiang X., Liu J., Zhao H. (2021). FDA Approval Summary: Atezolizumab Plus Bevacizumab for the Treatment of Patients with Advanced Unresectable or Metastatic Hepatocellular Carcinoma. Clin. Cancer Res..

[24] Psilopatis I., Damaskos C., Garmpi A., Sarantis P., Koustas E., Antoniou E.A., Dimitroulis D., Kouraklis G., Karamouzis M.V., Vrettou K. (2023). FDA-Approved Monoclonal Antibodies for Unresectable Hepatocellular Carcinoma: What Do We Know So Far?. Int. J. Mol. Sci..

[25] Vogel A., Cervantes A., Chau I., Daniele B., Llovet J.M., Meyer T., Nault J.C., Neumann U., Ricke J., Sangro B. (2018). Hepatocellular carcinoma: ESMO Clinical Practice Guidelines for diagnosis, treatment and follow-up. Ann. Oncol..

[26] Finn R.S., Qin S., Ikeda M., Galle P.R., Ducreux M., Kim T.Y., Kudo M., Breder V., Merle P., Kaseb A.O. (2020). Atezolizumab plus Bevacizumab in Unresectable Hepatocellular Carcinoma. N. Engl. J. Med..

[27] Vogrig A., Muniz-Castrillo S., Farina A., Honnorat J., Joubert B. (2022). How to diagnose and manage neurological toxicities of immune checkpoint inhibitors: An update. J. Neurol..

[28] Remash D., Prince D.S., McKenzie C., Strasser S.I., Kao S., Liu K. (2021). Immune checkpoint inhibitor-related hepatotoxicity: A review. World J. Gastroenterol..

[29] Chen R., Zhou M., Zhu F. (2022). Immune Checkpoint Inhibitors Related to Cardiotoxicity. J. Cardiovasc. Dev. Dis..

[30] Mattick J.S., Makunin I.V. (2006). Non-coding RNA. Hum. Mol. Genet..

[31] El-Daly S.M., Talaat R.M., Braoudaki M., Youness R.A., Cho W.C. (2023). Editorial: Recent breakthroughs in the decoding of circulating nucleic acids and their applications to human diseases. Front. Mol. Biosci..

[32] Youness R.A., Hafez H.M., Khallaf E., Assal R.A., Abdel Motaal A., Gad M.Z. (2019). The long noncoding RNA sONE represses triple-negative breast cancer aggressiveness through inducing the expression of miR-34a, miR-15a, miR-16, and let-7a. J. Cell Physiol..

[33] Selem N.A., Youness R.A., Gad M.Z. (2021). What is beyond LncRNAs in breast cancer: A special focus on colon cancer-associated Transcript-1 (CCAT-1). Noncoding RNA Res..

[34] Youness R.A., Assal R.A., Abdel Motaal A., Gad M.Z. (2018). A novel role of sONE/NOS3/NO signaling cascade in mediating hydrogen sulphide bilateral effects on triple negative breast cancer progression. Nitric Oxide.

[35] Mekky R.Y., El-Ekiaby N., El Sobky S.A., Elemam N.M., Youness R.A., El-Sayed M., Hamza M.T., Esmat G., Abdelaziz A.I. (2019). Epigallocatechin gallate (EGCG) and miR-548m reduce HCV entry through repression of CD81 receptor in HCV cell models. Arch. Virol..

[36] Nafea H., Youness R.A., Abou-Aisha K., Gad M.Z. (2021). LncRNA HEIH/miR-939-5p interplay modulates triple-negative breast cancer progression through NOS2-induced nitric oxide production. J. Cell Physiol..

[37] Fahmy S.A., Dawoud A., Zeinelabdeen Y.A., Kiriacos C.J., Daniel K.A., Eltahtawy O., Abdelhalim M.M., Braoudaki M., Youness R.A. (2022). Molecular Engines, Therapeutic Targets, and Challenges in Pediatric Brain Tumors: A Special Emphasis on Hydrogen Sulfide and RNA-Based Nano-Delivery. Cancers.

[38] Yan H., Bu P. (2021). Non-coding RNA in cancer. Essays Biochem..

[39] Matsui M., Corey D.R. (2017). Non-coding RNAs as drug targets. Nat. Rev. Drug Discov..

[40] Xu Z., Li P., Fan L., Wu M. (2018). The Potential Role of circRNA in Tumor Immunity Regulation and Immunotherapy. Front. Immunol..

[41] Dawoud A., Ihab Zakaria Z., Hisham Rashwan H., Braoudaki M., Youness R.A. (2023). Circular RNAs: New layer of complexity evading breast cancer heterogeneity. Noncoding RNA Res..

[42] Peng Z., Fang S., Jiang M., Zhao X., Zhou C., Gong Z. (2020). Circular RNAs: Regulatory functions in respiratory tract cancers. Clin. Chim. Acta.

[43] Wang F., Li X., Li Z., Wang S., Fan J. (2020). Functions of Circular RNAs in Regulating Adipogenesis of Mesenchymal Stem Cells. Stem Cells Int..

[44] Momen-Heravi F., Bala S. (2018). Emerging role of non-coding RNA in oral cancer. Cell Signal.

[45] Chen L., Shan G. (2021). CircRNA in cancer: Fundamental mechanism and clinical potential. Cancer Lett..

[46] Zhang X., Zhang Q., Zhang K., Wang F., Qiao X., Cui J. (2021). Circ SMARCA5 Inhibited Tumor Metastasis by Interacting with SND1 and Downregulating the YWHAB Gene in Cervical Cancer. Cell Transpl..

[47] El-Aziz M.K.A., Dawoud A., Kiriacos C.J., Fahmy S.A., Hamdy N.M., Youness R.A. (2023). Decoding hepatocarcinogenesis from a noncoding RNAs perspective. J. Cell Physiol..

[48] Zhang L., Xu X., Su X. (2020). Noncoding RNAs in cancer immunity: Functions, regulatory mechanisms, and clinical application. Mol. Cancer.

[49] Youness R.A., Gad A.Z., Sanber K., Ahn Y.J., Lee G.J., Khallaf E., Hafez H.M., Motaal A.A., Ahmed N., Gad M.Z. (2021). Targeting hydrogen sulphide signaling in breast cancer. J. Adv. Res..

[50] Papatsirou M., Artemaki P.I., Scorilas A., Kontos C.K. (2020). The role of circular RNAs in therapy resistance of patients with solid tumors. Per Med..

[51] Salzman J., Chen R.E., Olsen M.N., Wang P.L., Brown P.O. (2013). Cell-type specific features of circular RNA expression. PLoS Genet..

[52] Dragomir M., Calin G.A. (2018). Circular RNAs in Cancer—Lessons Learned From microRNAs. Front. Oncol..

[53] Li J., Sun D., Pu W., Wang J., Peng Y. (2020). Circular RNAs in Cancer: Biogenesis, Function, and Clinical Significance. Trends Cancer.

[54] Jeck W.R., Sharpless N.E. (2014). Detecting and characterizing circular RNAs. Nat. Biotechnol..

[55] Vo J.N., Cieslik M., Zhang Y., Shukla S., Xiao L., Zhang Y., Wu Y.M., Dhanasekaran S.M., Engelke C.G., Cao X. (2019). The Landscape of Circular RNA in Cancer. Cell.

[56] Jeck W.R., Sorrentino J.A., Wang K., Slevin M.K., Burd C.E., Liu J., Marzluff W.F., Sharpless N.E. (2013). Circular RNAs are abundant, conserved, and associated with ALU repeats. RNA.

[57] Zhang Y., Zhang X.O., Chen T., Xiang J.F., Yin Q.F., Xing Y.H., Zhu S., Yang L., Chen L.L. (2013). Circular intronic long noncoding RNAs. Mol. Cell.

[58] Geng X., Jia Y., Zhang Y., Shi L., Li Q., Zang A., Wang H. (2020). Circular RNA: Biogenesis, degradation, functions and potential roles in mediating resistance to anticarcinogens. Epigenomics.

[59] Zhang X.O., Dong R., Zhang Y., Zhang J.L., Luo Z., Zhang J., Chen L.L., Yang L. (2016). Diverse alternative back-splicing and alternative splicing landscape of circular RNAs. Genome Res..

[60] Wang M., Yu F., Li P. (2018). Circular RNAs: Characteristics, Function and Clinical Significance in Hepatocellular Carcinoma. Cancers.

[61] Bolha L., Ravnik-Glavac M., Glavac D. (2017). Circular RNAs: Biogenesis, Function, and a Role as Possible Cancer Biomarkers. Int. J. Genom..

[62] Papatsirou M., Artemaki P.I., Karousi P., Scorilas A., Kontos C.K. (2021). Circular RNAs: Emerging Regulators of the Major Signaling Pathways Involved in Cancer Progression. Cancers.

[63] Guo J.U., Agarwal V., Guo H., Bartel D.P. (2014). Expanded identification and characterization of mammalian circular RNAs. Genome Biol..

[64] Floris G., Zhang L., Follesa P., Sun T. (2017). Regulatory Role of Circular RNAs and Neurological Disorders. Mol. Neurobiol..

[65] Hansen T.B., Jensen T.I., Clausen B.H., Bramsen J.B., Finsen B., Damgaard C.K., Kjems J. (2013). Natural RNA circles function as efficient microRNA sponges. Nature.

[66] Shen T., Han M., Wei G., Ni T. (2015). An intriguing RNA species--perspectives of circularized RNA. Protein Cell.

[67] Conn S.J., Pillman K.A., Toubia J., Conn V.M., Salmanidis M., Phillips C.A., Roslan S., Schreiber A.W., Gregory P.A., Goodall G.J. (2015). The RNA binding protein quaking regulates formation of circRNAs. Cell.

[68] Errichelli L., Dini Modigliani S., Laneve P., Colantoni A., Legnini I., Capauto D., Rosa A., De Santis R., Scarfo R., Peruzzi G. (2017). FUS affects circular RNA expression in murine embryonic stem cell-derived motor neurons. Nat. Commun..

[69] Liang D., Tatomer D.C., Luo Z., Wu H., Yang L., Chen L.L., Cherry S., Wilusz J.E. (2017). The Output of Protein-Coding Genes Shifts to Circular RNAs When the Pre-mRNA Processing Machinery Is Limiting. Mol. Cell.

[70] Liang G., Yang Y., Niu G., Tang Z., Li K. (2017). Genome-wide profiling of Sus scrofa circular RNAs across nine organs and three developmental stages. DNA Res..

[71] Li P., Chen H., Chen S., Mo X., Li T., Xiao B., Yu R., Guo J. (2017). Circular RNA 0000096 affects cell growth and migration in gastric cancer. Br. J. Cancer.

[72] Huang S., Yang B., Chen B.J., Bliim N., Ueberham U., Arendt T., Janitz M. (2017). The emerging role of circular RNAs in transcriptome regulation. Genomics.

[73] Hansen T.B., Kjems J., Damgaard C.K. (2013). Circular RNA and miR-7 in cancer. Cancer Res..

[74] Yu L., Gong X.J., Sun L., Zhou Q.Y., Lu B.L., Zhu L.Y. (2016). The Circular RNA Cdr1as Act as an Oncogene in Hepatocellular Carcinoma through Targeting miR-7 Expression. PLoS ONE.

[75] Xu L., Zhang M., Zheng X., Yi P., Lan C., Xu M. (2017). The circular RNA ciRS-7 (Cdr1as) acts as a risk factor of hepatic microvascular invasion in hepatocellular carcinoma. J. Cancer Res. Clin. Oncol..

[76] Ren S., Xin Z., Xu Y., Xu J., Wang G. (2017). Construction and analysis of circular RNA molecular regulatory networks in liver cancer. Cell Cycle.

[77] Sun Y., Yang Z., Zheng B., Zhang X.H., Zhang M.L., Zhao X.S., Zhao H.Y., Suzuki T., Wen J.K. (2017). A Novel Regulatory Mechanism of Smooth Muscle alpha-Actin Expression by NRG-1/circACTA2/miR-548f-5p Axis. Circ. Res..

[78] Liu C., Yao M.D., Li C.P., Shan K., Yang H., Wang J.J., Liu B., Li X.M., Yao J., Jiang Q. (2017). Silencing Of Circular RNA-ZNF609 Ameliorates Vascular Endothelial Dysfunction. Theranostics.

[79] Rahmoon M.A., Youness R.A., Gomaa A.I., Hamza M.T., Waked I., El Tayebi H.M., Abdelaziz A.I. (2017). MiR-615-5p depresses natural killer cells cytotoxicity through repressing IGF-1R in hepatocellular carcinoma patients. Growth Factors.

[80] Zhong Z., Huang M., Lv M., He Y., Duan C., Zhang L., Chen J. (2017). Circular RNA MYLK as a competing endogenous RNA promotes bladder cancer progression through modulating VEGFA/VEGFR2 signaling pathway. Cancer Lett..

[81] Zhang H., Wang G.C., Ding C., Liu P., Wang R.K., Ding W.B., Tong D.K., Wu D.J., Li C., Wei Q. (2017). Increased circular RNA UBAP2 acts as a sponge of miR-143 to promote osteosarcoma progression. Oncotarget.

[82] Liang H.F., Zhang X.Z., Liu B.G., Jia G.T., Li W.L. (2017). Circular RNA circ-ABCB10 promotes breast cancer proliferation and progression through sponging miR-1271. Am. J. Cancer Res..

[83] Wang X.H., Fang L. (2018). Advances in circular RNAs and their roles in breast Cancer. J. Exp. Clin. Cancer Res..

[84] Yu T., Ran L., Zhao H., Yin P., Li W., Lin J., Mao H., Cai D., Ma Q., Pan X. (2021). Circular RNA circ-TNPO3 suppresses metastasis of GC by acting as a protein decoy for IGF2BP3 to regulate the expression of MYC and SNAIL. Mol. Ther. Nucleic Acids.

[85] Shaalan Y.M., Handoussa H., Youness R.A., Assal R.A., El-Khatib A.H., Linscheid M.W., El Tayebi H.M., Abdelaziz A.I. (2018). Destabilizing the interplay between miR-1275 and IGF2BPs by Tamarix articulata and quercetin in hepatocellular carcinoma. Nat. Prod. Res..

[86] Wang X., Liu S., Xu B., Liu Y., Kong P., Li C., Li B. (2021). circ-SIRT1 Promotes Colorectal Cancer Proliferation and EMT by Recruiting and Binding to eIF4A3. Anal. Cell Pathol..

[87] Huang A., Zheng H., Wu Z., Chen M., Huang Y. (2020). Circular RNA-protein interactions: Functions, mechanisms, and identification. Theranostics.

[88] Chen C., Zhang M., Zhang Y. (2020). Circ_0000079 Decoys the RNA-Binding Protein FXR1 to Interrupt Formation of the FXR1/PRCKI Complex and Decline Their Mediated Cell Invasion and Drug Resistance in NSCLC. Cell Transplant..

[89] Li Q., Wang Y., Wu S., Zhou Z., Ding X., Shi R., Thorne R.F., Zhang X.D., Hu W., Wu M. (2019). CircACC1 Regulates Assembly and Activation of AMPK Complex under Metabolic Stress. Cell Metab..

[90] Du W.W., Fang L., Yang W., Wu N., Awan F.M., Yang Z., Yang B.B. (2017). Induction of tumor apoptosis through a circular RNA enhancing Foxo3 activity. Cell Death Differ..

[91] Conn V.M., Hugouvieux V., Nayak A., Conos S.A., Capovilla G., Cildir G., Jourdain A., Tergaonkar V., Schmid M., Zubieta C. (2017). A circRNA from SEPALLATA3 regulates splicing of its cognate mRNA through R-loop formation. Nat. Plants.

[92] Xu X., Zhang J., Tian Y., Gao Y., Dong X., Chen W., Yuan X., Yin W., Xu J., Chen K. (2020). CircRNA inhibits DNA damage repair by interacting with host gene. Mol. Cancer.

[93] Li Z., Huang C., Bao C., Chen L., Lin M., Wang X., Zhong G., Yu B., Hu W., Dai L. (2015). Exon-intron circular RNAs regulate transcription in the nucleus. Nat. Struct. Mol. Biol..

[94] Pamudurti N.R., Bartok O., Jens M., Ashwal-Fluss R., Stottmeister C., Ruhe L., Hanan M., Wyler E., Perez-Hernandez D., Ramberger E. (2017). Translation of CircRNAs. Mol. Cell.

[95] Zhang M., Huang N., Yang X., Luo J., Yan S., Xiao F., Chen W., Gao X., Zhao K., Zhou H. (2018). A novel protein encoded by the circular form of the SHPRH gene suppresses glioma tumorigenesis. Oncogene.

[96] Zheng X., Chen L., Zhou Y., Wang Q., Zheng Z., Xu B., Wu C., Zhou Q., Hu W., Wu C. (2019). A novel protein encoded by a circular RNA circPPP1R12A promotes tumor pathogenesis and metastasis of colon cancer via Hippo-YAP signaling. Mol. Cancer.

[97] Liang W.C., Wong C.W., Liang P.P., Shi M., Cao Y., Rao S.T., Tsui S.K., Waye M.M., Zhang Q., Fu W.M. (2019). Translation of the circular RNA circbeta-catenin promotes liver cancer cell growth through activation of the Wnt pathway. Genome Biol..

[98] Di Timoteo G., Dattilo D., Centron-Broco A., Colantoni A., Guarnacci M., Rossi F., Incarnato D., Oliviero S., Fatica A., Morlando M. (2020). Modulation of circRNA Metabolism by m(6)A Modification. Cell Rep..

[99] Lee Y., Choe J., Park O.H., Kim Y.K. (2020). Molecular Mechanisms Driving mRNA Degradation by m(6)A Modification. Trends Genet..

[100] Yang Y., Fan X., Mao M., Song X., Wu P., Zhang Y., Jin Y., Yang Y., Chen L.L., Wang Y. (2017). Extensive translation of circular RNAs driven by N(6)-methyladenosine. Cell Res..

[101] Bird A. (2002). DNA methylation patterns and epigenetic memory. Genes. Dev..

[102] Lv J.F., Hu L., Zhuo W., Zhang C.M., Zhou H.H., Fan L. (2016). Epigenetic alternations and cancer chemotherapy response. Cancer Chemother. Pharmacol..

[103] Chen N., Zhao G., Yan X., Lv Z., Yin H., Zhang S., Song W., Li X., Li L., Du Z. (2018). A novel FLI1 exonic circular RNA promotes metastasis in breast cancer by coordinately regulating TET1 and DNMT1. Genome Biol..

[104] Margueron R., Reinberg D. (2011). The Polycomb complex PRC2 and its mark in life. Nature.

[105] Su M., Xiao Y., Tang J., Wu J., Ma J., Tian B., Zhou Y., Wang H., Yang D., Liao Q.J. (2018). Role of lncRNA and EZH2 Interaction/Regulatory Network in Lung Cancer. J. Cancer.

[106] Li B., Xie F., Zheng F.X., Jiang G.S., Zeng F.Q., Xiao X.Y. (2017). Overexpression of CircRNA BCRC4 regulates cell apoptosis and MicroRNA-101/EZH2 signaling in bladder cancer. Curr. Med. Sci..

[107] Qu D., Yan B., Xin R., Ma T. (2018). A novel circular RNA hsa_circ_0020123 exerts oncogenic properties through suppression of miR-144 in non-small cell lung cancer. Am. J. Cancer Res..

[108] Kristensen L.S., Andersen M.S., Stagsted L.V.W., Ebbesen K.K., Hansen T.B., Kjems J. (2019). The biogenesis, biology and characterization of circular RNAs. Nat. Rev. Genet..

[109] Zhang J., Chang Y., Xu L., Qin L. (2019). Elevated expression of circular RNA circ_0008450 predicts dismal prognosis in hepatocellular carcinoma and regulates cell proliferation, apoptosis, and invasion via sponging miR-548p. J. Cell Biochem..

[110] Yu J., Yang M., Zhou B., Luo J., Zhang Z., Zhang W., Yan Z. (2019). CircRNA-104718 acts as competing endogenous RNA and promotes hepatocellular carcinoma progression through microRNA-218-5p/TXNDC5 signaling pathway. Clin. Sci..

[111] Xie B., Zhao Z., Liu Q., Wang X., Ma Z., Li H. (2019). CircRNA has_circ_0078710 acts as the sponge of microRNA-31 involved in hepatocellular carcinoma progression. Gene.

[112] Gong Y., Mao J., Wu D., Wang X., Li L., Zhu L., Song R. (2018). Circ-ZEB1.33 promotes the proliferation of human HCC by sponging miR-200a-3p and upregulating CDK6. Cancer Cell Int..

[113] Guan Z., Tan J., Gao W., Li X., Yang Y., Li X., Li Y., Wang Q. (2018). Circular RNA hsa_circ_0016788 regulates hepatocellular carcinoma tumorigenesis through miR-486/CDK4 pathway. J. Cell Physiol..

[114] Wei X., Zheng W., Tian P., He Y., Liu H., Peng M., Li X., Liu X. (2020). Oncogenic hsa_circ_0091581 promotes the malignancy of HCC cell through blocking miR-526b from degrading c-MYC mRNA. Cell Cycle.

[115] Liu B., Yang G., Wang X., Liu J., Lu Z., Wang Q., Xu B., Liu Z., Li J. (2020). CircBACH1 (hsa_circ_0061395) promotes hepatocellular carcinoma growth by regulating p27 repression via HuR. J. Cell Physiol..

[116] Pu J., Wang J., Li W., Lu Y., Wu X., Long X., Luo C., Wei H. (2020). hsa_circ_0000092 promotes hepatocellular carcinoma progression through up-regulating HN1 expression by binding to microRNA-338-3p. J. Cell Mol. Med..

[117] Yang G., Wang X., Liu B., Lu Z., Xu Z., Xiu P., Liu Z., Li J. (2019). circ-BIRC6, a circular RNA, promotes hepatocellular carcinoma progression by targeting the miR-3918/Bcl2 axis. Cell Cycle.

[118] Chen W., Quan Y., Fan S., Wang H., Liang J., Huang L., Chen L., Liu Q., He P., Ye Y. (2020). Exosome-transmitted circular RNA hsa_circ_0051443 suppresses hepatocellular carcinoma progression. Cancer Lett..

[119] Song C., Li D., Liu H., Sun H., Liu Z., Zhang L., Hu Y. (2019). The competing endogenous circular RNA ADAMTS14 suppressed hepatocellular carcinoma progression through regulating microRNA-572/regulator of calcineurin 1. J. Cell Physiol..

[120] Liu Z., Yu Y., Huang Z., Kong Y., Hu X., Xiao W., Quan J., Fan X. (2019). CircRNA-5692 inhibits the progression of hepatocellular carcinoma by sponging miR-328-5p to enhance DAB2IP expression. Cell Death Dis..

[121] Dong W., Dai Z.H., Liu F.C., Guo X.G., Ge C.M., Ding J., Liu H., Yang F. (2019). The RNA-binding protein RBM3 promotes cell proliferation in hepatocellular carcinoma by regulating circular RNA SCD-circRNA 2 production. EBioMedicine.

[122] Wang L., Long H., Zheng Q., Bo X., Xiao X., Li B. (2019). Circular RNA circRHOT1 promotes hepatocellular carcinoma progression by initiation of NR2F6 expression. Mol. Cancer.

[123] Huang X.Y., Huang Z.L., Huang J., Xu B., Huang X.Y., Xu Y.H., Zhou J., Tang Z.Y. (2020). Exosomal circRNA-100338 promotes hepatocellular carcinoma metastasis via enhancing invasiveness and angiogenesis. J. Exp. Clin. Cancer Res..

[124] Ding Z., Guo L., Deng Z., Li P. (2020). Circ-PRMT5 enhances the proliferation, migration and glycolysis of hepatoma cells by targeting miR-188-5p/HK2 axis. Ann. Hepatol..

[125] Li Q., Pan X., Zhu D., Deng Z., Jiang R., Wang X. (2019). Circular RNA MAT2B Promotes Glycolysis and Malignancy of Hepatocellular Carcinoma Through the miR-338-3p/PKM2 Axis Under Hypoxic Stress. Hepatology.

[126] Hu Z.Q., Zhou S.L., Li J., Zhou Z.J., Wang P.C., Xin H.Y., Mao L., Luo C.B., Yu S.Y., Huang X.W. (2020). Circular RNA Sequencing Identifies CircASAP1 as a Key Regulator in Hepatocellular Carcinoma Metastasis. Hepatology.

[127] Zhang P.F., Gao C., Huang X.Y., Lu J.C., Guo X.J., Shi G.M., Cai J.B., Ke A.W. (2020). Cancer cell-derived exosomal circUHRF1 induces natural killer cell exhaustion and may cause resistance to anti-PD1 therapy in hepatocellular carcinoma. Mol. Cancer.

[128] Xiong D., He R., Dang Y., Wu H., Feng Z., Chen G. (2020). The Latest Overview of circRNA in the Progression, Diagnosis, Prognosis, Treatment, and Drug Resistance of Hepatocellular Carcinoma. Front. Oncol..

[129] Wei Y., Chen X., Liang C., Ling Y., Yang X., Ye X., Zhang H., Yang P., Cui X., Ren Y. (2020). A Noncoding Regulatory RNAs Network Driven by Circ-CDYL Acts Specifically in the Early Stages Hepatocellular Carcinoma. Hepatology.

[130] Zhai Z., Fu Q., Liu C., Zhang X., Jia P., Xia P., Liu P., Liao S., Qin T., Zhang H. (2019). Emerging Roles Of hsa-circ-0046600 Targeting The miR-640/HIF-1alpha Signalling Pathway In The Progression Of HCC. Onco Targets Ther..

[131] Zou H., Xu X., Luo L., Zhang Y., Luo L., Yao Y., Xiang G., Huang X., Wang G. (2019). Hsa_circ_0101432 promotes the development of hepatocellular carcinoma (HCC) by adsorbing miR-1258 and miR-622. Cell Cycle.

[132] Fu X., Zhang J., He X., Yan X., Wei J., Huang M., Liu Y., Lin J., Hu H., Liu L. (2020). Circular RNA MAN2B2 promotes cell proliferation of hepatocellular carcinoma cells via the miRNA-217/MAPK1 axis. J. Cancer.

[133] Wang G., Liu W., Zou Y., Wang G., Deng Y., Luo J., Zhang Y., Li H., Zhang Q., Yang Y. (2019). Three isoforms of exosomal circPTGR1 promote hepatocellular carcinoma metastasis via the miR449a-MET pathway. EBioMedicine.

[134] Zhang H., Deng T., Ge S., Liu Y., Bai M., Zhu K., Fan Q., Li J., Ning T., Tian F. (2019). Exosome circRNA secreted from adipocytes promotes the growth of hepatocellular carcinoma by targeting deubiquitination-related USP7. Oncogene.

[135] Su Y., Lv X., Yin W., Zhou L., Hu Y., Zhou A., Qi F. (2019). CircRNA Cdr1as functions as a competitive endogenous RNA to promote hepatocellular carcinoma progression. Aging.

[136] Zhu Y., Liu Y., Xiao B., Cai H., Liu M., Ma L., Yin H., Wang F. (2019). The circular RNA PVT1/miR-203/HOXD3 pathway promotes the progression of human hepatocellular carcinoma. Biol. Open.

[137] Bu N., Dong Z., Zhang L., Zhu W., Wei F., Zheng S. (2020). CircPVT1 Regulates Cell Proliferation, Apoptosis and Glycolysis in Hepatocellular Carcinoma via miR-377/TRIM23 Axis. Cancer Manag. Res..

[138] Lin T., Dai Y., Guo X., Chen W., Zhao J., Cao L., Wu Z. (2019). Silencing Of hsa_circ_0008450 Represses Hepatocellular Carcinoma Progression Through Regulation Of microRNA-214-3p/EZH2 Axis. Cancer Manag. Res..

[139] Zhan W., Liao X., Chen Z., Li L., Tian T., Yu L., Wang W., Hu Q. (2020). Circular RNA hsa_circRNA_103809 promoted hepatocellular carcinoma development by regulating miR-377-3p/FGFR1/ERK axis. J. Cell Physiol..

[140] Li Z., Hu Y., Zeng Q., Wang H., Yan J., Li H., Yu Z. (2019). Circular RNA MYLK promotes hepatocellular carcinoma progression by increasing Rab23 expression by sponging miR-362-3p. Cancer Cell Int..

[141] Li Y., Zang H., Zhang X., Huang G. (2020). Exosomal Circ-ZNF652 Promotes Cell Proliferation, Migration, Invasion and Glycolysis in Hepatocellular Carcinoma via miR-29a-3p/GUCD1 Axis. Cancer Manag. Res..

[142] Pan H., Tang L., Jiang H., Li X., Wang R., Gao J., Li Q. (2019). Enhanced expression of circ_0000267 in hepatocellular carcinoma indicates poor prognosis and facilitates cell progression by sponging miR-646. J. Cell Biochem..

[143] Wang W., Li Y., Li X., Liu B., Han S., Li X., Zhang B., Li J., Sun S. (2020). Circular RNA circ-FOXP1 induced by SOX9 promotes hepatocellular carcinoma progression via sponging miR-875-3p and miR-421. Biomed. Pharmacother..

[144] Zhang X., Xu Y., Qian Z., Zheng W., Wu Q., Chen Y., Zhu G., Liu Y., Bian Z., Xu W. (2018). circRNA_104075 stimulates YAP-dependent tumorigenesis through the regulation of HNF4a and may serve as a diagnostic marker in hepatocellular carcinoma. Cell Death Dis..

[145] Cao S., Wang G., Wang J., Li C., Zhang L. (2019). Hsa_circ_101280 promotes hepatocellular carcinoma by regulating miR-375/JAK2. Immunol. Cell Biol..

[146] Li S., Gu H., Huang Y., Peng Q., Zhou R., Yi P., Chen R., Huang Z., Hu X., Huang Y. (2018). Circular RNA 101368/miR-200a axis modulates the migration of hepatocellular carcinoma through HMGB1/RAGE signaling. Cell Cycle.

[147] Bai N., Peng E., Qiu X., Lyu N., Zhang Z., Tao Y., Li X., Wang Z. (2018). circFBLIM1 act as a ceRNA to promote hepatocellular cancer progression by sponging miR-346. J. Exp. Clin. Cancer Res..

[148] Cai H., Hu B., Ji L., Ruan X., Zheng Z. (2018). Hsa_circ_0103809 promotes cell proliferation and inhibits apoptosis in hepatocellular carcinoma by targeting miR-490-5p/SOX2 signaling pathway. Am. J. Transl. Res..

[149] Wang B., Chen H., Zhang C., Yang T., Zhao Q., Yan Y., Zhang Y., Xu F. (2018). Effects of hsa_circRBM23 on Hepatocellular Carcinoma Cell Viability and Migration as Produced by Regulating miR-138 Expression. Cancer Biother. Radiopharm..

[150] Li M.F., Li Y.H., He Y.H., Wang Q., Zhang Y., Li X.F., Meng X.M., Huang C., Li J. (2018). Emerging roles of hsa_circ_0005075 targeting miR-431 in the progress of HCC. Biomed. Pharmacother..

[151] Bai N., Peng E., Xia F., Wang D., Li X., Li X. (2019). CircABCC2 Regulates Hepatocellular Cancer Progression by Decoying MiR-665. J. Cancer.

[152] Huang X.Y., Huang Z.L., Zhang P.B., Huang X.Y., Huang J., Wang H.C., Xu B., Zhou J., Tang Z.Y. (2019). CircRNA-100338 Is Associated With mTOR Signaling Pathway and Poor Prognosis in Hepatocellular Carcinoma. Front. Oncol..

[153] Ji C., Hong X., Lan B., Lin Y., He Y., Chen J., Liu X., Ye W., Mo Z., She Z. (2021). Circ_0091581 Promotes the Progression of Hepatocellular Carcinoma Through Targeting miR-591/FOSL2 Axis. Dig. Dis. Sci..

[154] Sun P., Fan X., Hu X., Fu X., Wei Q., Zang Y. (2019). circPCNX and Pecanex Promote Hepatocellular Carcinoma Cell Viability by Inhibiting miR-506. Cancer Manag. Res..

[155] Li Z., Liu Y., Yan J., Zeng Q., Hu Y., Wang H., Li H., Li J., Yu Z. (2020). Circular RNA hsa_circ_0056836 functions an oncogenic gene in hepatocellular carcinoma through modulating miR-766-3p/FOSL2 axis. Aging.

[156] Zhao M., Dong G., Meng Q., Lin S., Li X. (2020). Circ-HOMER1 enhances the inhibition of miR-1322 on CXCL6 to regulate the growth and aggressiveness of hepatocellular carcinoma cells. J. Cell Biochem..

[157] Liu M., Guo B., Zhang G., Qi H. (2023). Circ_0091579 Knockdown Inhibited HCC Proliferation and Glutamine Metabolism Through miR-1270/YAP1 Axis. Biochem. Genet..

[158] Mao Y., Ding Z., Jiang M., Yuan B., Zhang Y., Zhang X. (2022). Circ_0091579 exerts an oncogenic role in hepatocellular carcinoma via mediating miR-136-5p/TRIM27. Biomed. J..

[159] Zhang D., Zhang Y., Zhang X., Zhai H., Sun X., Li Y. (2022). Circ_0091579 Serves as a Tumor-Promoting Factor in Hepatocellular Carcinoma Through miR-1225-5p/PLCB1 Axis. Dig. Dis. Sci..

[160] Ding B., Fan W., Lou W. (2020). hsa_circ_0001955 Enhances In Vitro Proliferation, Migration, and Invasion of HCC Cells through miR-145-5p/NRAS Axis. Mol. Ther. Nucleic Acids.

[161] Yao Z., Xu R., Yuan L., Xu M., Zhuang H., Li Y., Zhang Y., Lin N. (2019). Circ_0001955 facilitates hepatocellular carcinoma (HCC) tumorigenesis by sponging miR-516a-5p to release TRAF6 and MAPK11. Cell Death Dis..

[162] Li X., Lv J., Hou L., Guo X. (2022). Circ_0001955 Acts as a miR-646 Sponge to Promote the Proliferation, Metastasis and Angiogenesis of Hepatocellular Carcinoma. Dig. Dis. Sci..

[163] Zhang P.F., Wei C.Y., Huang X.Y., Peng R., Yang X., Lu J.C., Zhang C., Gao C., Cai J.B., Gao P.T. (2019). Circular RNA circTRIM33-12 acts as the sponge of MicroRNA-191 to suppress hepatocellular carcinoma progression. Mol. Cancer.

[164] Wang Z., Zhao Y., Wang Y., Jin C. (2019). Circular RNA circHIAT1 inhibits cell growth in hepatocellular carcinoma by regulating miR-3171/PTEN axis. Biomed. Pharmacother..

[165] Chen Z., Zuo X., Pu L., Zhang Y., Han G., Zhang L., Wu J., Wang X. (2019). circLARP4 induces cellular senescence through regulating miR-761/RUNX3/p53/p21 signaling in hepatocellular carcinoma. Cancer Sci..

[166] Wang J., Tan Q., Wang W., Yu J. (2020). Mechanism of the Regulatory Effect of Overexpression of circMTO1 on Proliferation and Apoptosis of Hepatoma Cells via miR-9-5p/NOX4 Axis. Cancer Manag. Res..

[167] Guo X., Wang Z., Deng X., Lu Y., Huang X., Lin J., Lan X., Su Q., Wang C. (2022). Circular RNA CircITCH (has-circ-0001141) suppresses hepatocellular carcinoma (HCC) progression by sponging miR-184. Cell Cycle.

[168] Chen X., Li H.D., Bu F.T., Li X.F., Chen Y., Zhu S., Wang J.N., Chen S.Y., Sun Y.Y., Pan X.Y. (2020). Circular RNA circFBXW4 suppresses hepatic fibrosis via targeting the miR-18b-3p/FBXW7 axis. Theranostics.

[169] Matboli M., Hassan M.K., Ali M.A., Mansour M.T., Elsayed W., Atteya R., Aly H.S., Meteini M.E., Elghazaly H., El-Khamisy S. (2022). Impact of circ-0000221 in the Pathogenesis of Hepatocellular via Modulation of miR-661-PTPN11 mRNA Axis. Pharmaceutics.

[170] Shen H., Li H., Zhou J. (2022). Circular RNA hsa_circ_0032683 inhibits the progression of hepatocellular carcinoma by sponging microRNA-338-5p. Bioengineered.

[171] Xu Z.X., Li J.Z., Li Q., Xu M.Y., Li H.Y. (2022). CircRNA608-microRNA222-PINK1 axis regulates the mitophagy of hepatic stellate cells in NASH related fibrosis. Biochem. Biophys. Res. Commun..

[172] Ji D., Chen G.F., Wang J.C., Ji S.H., Wu X.W., Lu X.J., Chen J.L., Li J.T. (2020). Hsa_circ_0070963 inhibits liver fibrosis via regulation of miR-223-3p and LEMD3. Aging.

[173] Zhu P., Liang H., Huang X., Zeng Q., Liu Y., Lv J., Ming L. (2020). Circular RNA Hsa_circ_0004018 Inhibits Wnt/beta-Catenin Signaling Pathway by Targeting microRNA-626/DKK3 in Hepatocellular Carcinoma. Onco Targets Ther..

[174] van den Bulk J., Verdegaal E.M., de Miranda N.F. (2018). Cancer immunotherapy: Broadening the scope of targetable tumours. Open Biol..

[175] Feins S., Kong W., Williams E.F., Milone M.C., Fraietta J.A. (2019). An introduction to chimeric antigen receptor (CAR) T-cell immunotherapy for human cancer. Am. J. Hematol..

[176] Liu J., Fu M.Y., Wang M.N., Wan D.D., Wei Y.Q., Wei X.W. (2022). Cancer vaccines as promising immuno-therapeutics: Platforms and current progress. J. Hematol. Oncol..

[177] Kaufman H.L., Kohlhapp F.J., Zloza A. (2015). Oncolytic viruses: A new class of immunotherapy drugs. Nat. Rev. Drug Discov..

[178] Carlino M.S., Larkin J., Long G.V. (2021). Immune checkpoint inhibitors in melanoma. Lancet.

[179] Koerner J., Horvath D., Herrmann V.L., MacKerracher A., Gander B., Yagita H., Rohayem J., Groettrup M. (2021). PLGA-particle vaccine carrying TLR3/RIG-I ligand Riboxxim synergizes with immune checkpoint blockade for effective anti-cancer immunotherapy. Nat. Commun..

[180] Ahmed Youness R., Amr Assal R., Mohamed Ezzat S., Zakaria Gad M., Abdel Motaal A. (2020). A methoxylated quercetin glycoside harnesses HCC tumor progression in a TP53/miR-15/miR-16 dependent manner. Nat. Prod. Res..

[181] Li S., Zhang Z.B., Lai W.F., Cui L., Zhu X. (2020). How to overcome the side effects of tumor immunotherapy. Biomed. Pharmacother..

[182] Galli F., Aguilera J.V., Palermo B., Markovic S.N., Nistico P., Signore A. (2020). Relevance of immune cell and tumor microenvironment imaging in the new era of immunotherapy. J. Exp. Clin. Cancer Res..

[183] Mandlik D.S., Mandlik S.K., Choudhary H.B. (2023). Immunotherapy for hepatocellular carcinoma: Current status and future perspectives. World J. Gastroenterol..

[184] Schwarzenbach H., Hoon D.S., Pantel K. (2011). Cell-free nucleic acids as biomarkers in cancer patients. Nat. Rev. Cancer.

[185] Neves A.F., Dias-Oliveira J.D., Araujo T.G., Marangoni K., Goulart L.R. (2013). Prostate cancer antigen 3 (PCA3) RNA detection in blood and tissue samples for prostate cancer diagnosis. Clin. Chem. Lab. Med..

[186] Abdallah R.M., Elkhouly A.M., Soliman R.A., El Mechawy N., El Sebaei A., Motaal A.A., El-Askary H., Youness R.A., Assal R.A. (2022). Hindering the Synchronization Between miR-486-5p and H19 lncRNA by Hesperetin Halts Breast Cancer Aggressiveness Through Tuning ICAM-1. Anticancer. Agents Med. Chem..

[187] Abdel-Latif M., Riad A., Soliman R.A., Elkhouly A.M., Nafae H., Gad M.Z., Motaal A.A., Youness R.A. (2022). MALAT-1/p53/miR-155/miR-146a ceRNA circuit tuned by methoxylated quercitin glycoside alters immunogenic and oncogenic profiles of breast cancer. Mol. Cell Biochem..

[188] El Kilany F.H., Youness R.A., Assal R.A., Gad M.Z. (2021). miR-744/eNOS/NO axis: A novel target to halt triple negative breast cancer progression. Breast Dis..

[189] Dawoud A., Youness R.A., Nafea H., Manie T., Abdel-Kader R.M., Gad M. (2023). 26P 3MST: A potential workhorse in H2S signaling trimmed by microRNA-548 in breast cancer. ESMO Open.

[190] Soliman R., Youness R.A., El-Shazly M., Handoussa H., Gad M. (2019). Regulatory interacting network between the immunomodulatory non-coding RNAs: miR-17-5p, MALAT1 and H19 lncRNAs in modulating the tumour microenvironment in TNBC. Ann. Oncol..

[191] Awad A.R., Youness R.A., Ibrahim M., Motaal A.A., El-Askary H.I., Assal R.A., Gad M.Z. (2021). An acetylated derivative of vitexin halts MDA-MB-231 cellular progression and improves its immunogenic profile through tuning miR- 20a-MICA/B axis. Nat. Prod. Res..

[192] Arita T., Ichikawa D., Konishi H., Komatsu S., Shiozaki A., Shoda K., Kawaguchi T., Hirajima S., Nagata H., Kubota T. (2013). Circulating long non-coding RNAs in plasma of patients with gastric cancer. Anticancer. Res..

[193] Yu L.L., Xiao Q., Yu B., Lv Q.L., Liu Z.Q., Yin J.Y. (2023). CircRNAs in tumor immunity and immunotherapy: Perspectives from innate and adaptive immunity. Cancer Lett..

[194] Cheng H., Sun G., Chen H., Li Y., Han Z., Li Y., Zhang P., Yang L., Li Y. (2019). Trends in the treatment of advanced hepatocellular carcinoma: Immune checkpoint blockade immunotherapy and related combination therapies. Am. J. Cancer Res..

[195] Huang X.Y., Zhang P.F., Wei C.Y., Peng R., Lu J.C., Gao C., Cai J.B., Yang X., Fan J., Ke A.W. (2020). Circular RNA circMET drives immunosuppression and anti-PD1 therapy resistance in hepatocellular carcinoma via the miR-30-5p/snail/DPP4 axis. Mol. Cancer.

[196] Chen Z.Q., Zuo X.L., Cai J., Zhang Y., Han G.Y., Zhang L., Ding W.Z., Wu J.D., Wang X.H. (2023). Hypoxia-associated circPRDM4 promotes immune escape via HIF-1alpha regulation of PD-L1 in hepatocellular carcinoma. Exp. Hematol. Oncol..

[197] Cai J., Chen Z., Zhang Y., Wang J., Zhang Z., Wu J., Mao J., Zuo X. (2022). CircRHBDD1 augments metabolic rewiring and restricts immunotherapy efficacy via m(6)A modification in hepatocellular carcinoma. Mol. Ther. Oncolytics.

[198] Lu J.C., Zhang P.F., Huang X.Y., Guo X.J., Gao C., Zeng H.Y., Zheng Y.M., Wang S.W., Cai J.B., Sun Q.M. (2021). Amplification of spatially isolated adenosine pathway by tumor-macrophage interaction induces anti-PD1 resistance in hepatocellular carcinoma. J. Hematol. Oncol..

[199] Hu Z., Chen G., Zhao Y., Gao H., Li L., Yin Y., Jiang J., Wang L., Mang Y., Gao Y. (2023). Exosome-derived circCCAR1 promotes CD8 + T-cell dysfunction and anti-PD1 resistance in hepatocellular carcinoma. Mol. Cancer.

[200] Tanaka A., Sakaguchi S. (2017). Regulatory T cells in cancer immunotherapy. Cell Res..

[201] Zheng C., Zheng L., Yoo J.K., Guo H., Zhang Y., Guo X., Kang B., Hu R., Huang J.Y., Zhang Q. (2017). Landscape of Infiltrating T Cells in Liver Cancer Revealed by Single-Cell Sequencing. Cell.

[202] Huang M., Huang X., Huang N. (2022). Exosomal circGSE1 promotes immune escape of hepatocellular carcinoma by inducing the expansion of regulatory T cells. Cancer Sci..

[203] Huntington N.D., Cursons J., Rautela J. (2020). The cancer-natural killer cell immunity cycle. Nat. Rev. Cancer.

[204] Ma Y., Zhang C., Zhang B., Yu H., Yu Q. (2019). circRNA of AR-suppressed PABPC1 91 bp enhances the cytotoxicity of natural killer cells against hepatocellular carcinoma via upregulating UL16 binding protein 1. Oncol. Lett..

